# Zinc Transporters of the LIV-1 Subfamily in Various Cancers: Molecular Insights and Research Priorities for Saudi Arabia

**DOI:** 10.3390/ijms26168080

**Published:** 2025-08-21

**Authors:** Ahmed M. Alzahrani, Kathryn M. Taylor

**Affiliations:** 1 Department of Biochemistry, Al-Qunfudah Medical College, Umm Al-Qura University, Makkah 24382, Saudi Arabia; 2 School of Pharmacy and Pharmaceutical Sciences, Cardiff University, Cardiff CF10 3NB, UK

**Keywords:** zinc, zinc transporters, breast cancer, colorectal cancer, Saudi Arabia, prognostic biomarkers, cancer therapy, ZIP4, ZIP7, ZIP6

## Abstract

Zinc is an essential trace element involved in critical physiological functions such as gene expression, immune regulation, and cellular proliferation. This review explores the link between zinc homeostasis and cancer, with a specific focus on LIV-1 zinc transporters and their potential relevance to cancer research and treatment priorities in Saudi Arabia, as informed by global data. Zinc homeostasis is maintained by two major transporter families: ZIP (SLC39A) and ZnT (SLC30A). The dysregulation of specific ZIP transporters, particularly ZIP4, ZIP7, ZIP6, and ZIP10, has been implicated in cancer progression. Bioinformatic analyses revealed the significant overexpression of ZIP4, ZIP7, and ZIP6 in breast cancer and ZIP4 in colorectal cancer, which are the most common cancers among Saudi women and men, respectively. Notably, ZIP4 and ZIP7 upregulation correlated with poorer clinical outcomes, whereas ZIP6 was positively associated with survival in breast cancer. These findings underscore the potential of zinc transporters as prognostic biomarkers and therapeutic targets. Despite the substantial global evidence, research on zinc transporters in the Saudi population remains limited. Considering the Kingdom’s rising cancer burden and unique genetic, environmental, and dietary factors, understanding zinc metabolism in this context is important. Targeted research may support precision medicine strategies and improve outcomes in line with Saudi Arabia’s healthcare transformation goals.

## 1. Introduction

Zinc is an essential trace element in the human body. Zn plays a central role in numerous physiological functions, including reproduction, protection of cells from oxidative species, immune function, and cell growth and division [1,2]. Moreover, Zn acts as a structural, catalytic, and co-catalytic component of several proteins, enzymes, and transcription factors. Approximately 10% of proteins in the human body are zinc-bound proteins [3]. More than 300 enzymes require Zn for their activity, and over 2000 transcription factors, including gene expression, depend on Zn for integrity and facilitate binding to DNA [4]. Thus, Zn is an indispensable micronutrient required for many cellular processes, such as gene transcription, protein synthesis, cell growth, and division.

Zinc is an important trace element that plays a vital role in sustaining the integrity and functionality of skeletal development [5], immune response [6], endocrine pathways [7], neurological function [8], and sexual health [9].

For example, Zn is a key component in the mineralisation of bone tissue, contributing to structural integrity, supporting collagen matrix synthesis, and regulating bone turnover [5].

Moreover, Zn influences the immune system by regulating the intracellular signalling pathways involved in antibody production, lymphocyte differentiation, and inflammatory responses in both innate and adaptive immune cells [6,10]. Therefore, zinc is essential for maintaining immune competence and homeostasis. Zinc is also important for the proper functioning of the endocrine system.

For instance, the synthesis of thyroid hormones, which regulate many physiological functions, such as metabolism, body temperature, and heart rate, is regulated by zinc through its modulation of transcription factors required for hormone production [11]. This role is further supported by evidence showing reduced thyroid hormone levels during zinc deficiency as observed in the decreased level of thyroid hormones during zinc deficiency. Unsurprisingly, zinc supplementation enhances hormone synthesis and restores hormonal balance [12].

Additionally, zinc is important for insulin structure and function. Therefore, it is not surprising that zinc levels are increased in pancreatic beta cells compared to other cells in the body [13], indicating its critical role in insulin biosynthesis and regulation. Transporters, which facilitate zinc movement across cellular compartments, have also been shown to play a role in maintaining insulin release by modulating the signalling pathway between the pancreas and liver, ultimately leading to glycaemic control within the skeletal muscle [14,15].

In the central nervous system (CNS), zinc plays a critical role in the proper functioning of nerve cells, particularly through zinc-dependent enzymes and signalling pathways. Zinc has also been shown to inhibit GABA-A receptors, thereby decreasing their inhibitory effects [8,16]. As a result, alterations in zinc levels can strongly affect CNS function, contributing to conditions such as Alzheimer’s disease.

Finally, zinc is a key driver of sexual health and is essential for improving fertility in males. Many studies have demonstrated a strong relationship between zinc deficiency and impaired reproduction in males, indicating the critical role of zinc in male reproductive health [17]. Notably, prostate tissue contains the highest concentration of zinc in the male body, suggesting its significance in seminal plasma composition and sperm viability. Additionally, GPR39, a zinc-sensing receptor located in the sperm tail, has been observed to be triggered by extracellular zinc, promoting sperm motility and acrosomal exocytosis [18]. Collectively, these examples reflect the significant role of zinc in maintaining health.

Despite the extensive global literature highlighting the critical role of zinc in human health, research on zinc homeostasis remains significantly unexplored in Saudi Arabia. Several factors make the Saudi context particularly relevant to studies on zinc metabolism. Traditional Saudi diets are often high in rice and legumes [19], which are rich in phytates, compounds that inhibit zinc absorption [20]. This concern is supported by a recent large national study that reported widespread micronutrient deficiencies, including zinc, among Saudi adults [21]. Given the Kingdom’s unique dietary patterns, genetic background, and environmental exposures, it is crucial to investigate how these factors may influence zinc metabolism in Saudi Arabia. Addressing this research gap by applying insights from global datasets can help develop regionally informed strategies to improve disease prevention, diagnosis, and treatment.

## 2. Zinc Homeostasis

The human body contains approximately 3 g of zinc, with the highest concentrations found in the skeletal muscle and bone. Zinc homeostasis is tightly regulated by absorption, redistribution, and excretion to maintain appropriate zinc levels within cells [2,4]. At the cellular level, zinc homeostasis is maintained by the coordinated activities of zinc transporters and zinc-binding proteins.

The two main families of zinc transporters are ZIP (SLC39A) and ZnT (SLC30A). The ZIP family encompasses 14 members in humans (ZIP1-14) and imports zinc from either the extracellular space or cellular zinc stores into the cytoplasm [22].

**Figure 1:**
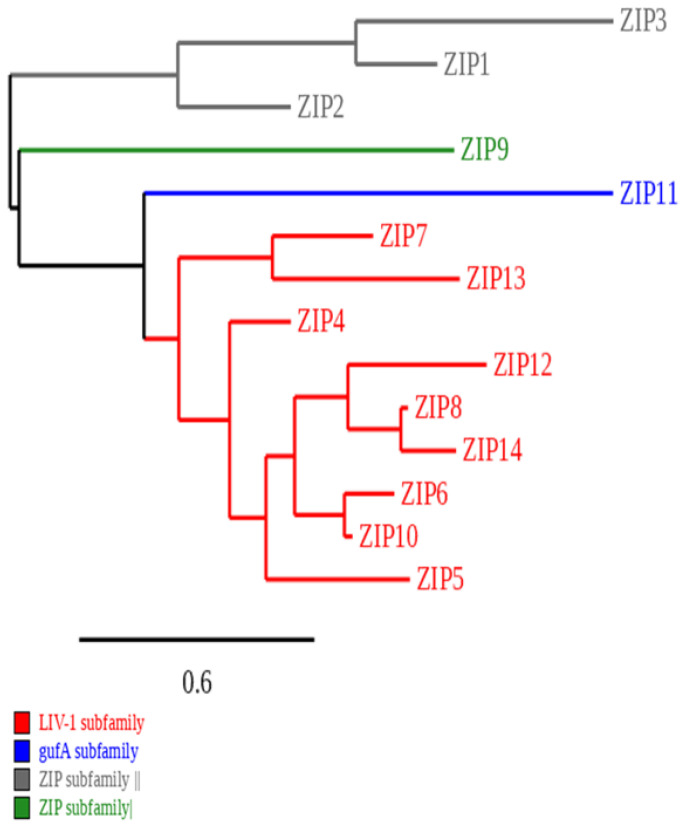
Phylogenetic tree of the human ZIP family of zinc transporters. This diagram demonstrates the categorisation of ZIP transporters according to the phylogenetic tree [23]. The horizontal axis of the phylogram is measured by genetic alterations, and the degree of these changes is illustrated by the scale shown at the bottom of the bar. The sequences of ZIP proteins were retrieved in FASTA format using the NCBI database, and the tree was constructed using the Phylogeny.fr web service [24].

ZIP transporters are divided into four subfamilies based on their structural and functional characteristics (Figure 1). Importantly, the LIV-1 subfamily of ZIP transporters (including ZIP4, ZIP5, ZIP6, ZIP7, ZIP8, ZIP10, ZIP12, ZIP13, and ZIP14) has been implicated in a range of diseases, particularly cancer, where altered expression can influence zinc homeostasis and promote oncogenic signalling [22]. In contrast, SLC30A or ZnT transporters have 10 human members (ZnT1 to ZnT10) and function to reduce the cytosolic concentration of zinc by mobilising it into the extracellular space or intracellular compartments.

In addition to zinc transporters, zinc-binding proteins, such as metallothioneins, participate in cellular zinc homeostasis by buffering cytosolic free zinc under physiological conditions and regulating the storage and release of intracellular zinc [25,26].

## 3. Zinc Dysregulation in Disease

Cellular zinc imbalance has been implicated in various diseases, including diabetes mellitus [14], inflammatory conditions [27], and cancer [28]. In diabetes, zinc plays a significant role in the formation of insulin hexameric units, which are the storage forms of insulin in beta cells. Furthermore, zinc affects insulin signalling pathways and glucose uptake in adipocytes. Therefore, zinc deficiency can impair insulin secretion and reduce glucose uptake by cells, leading to insulin resistance [13,14]. Accordingly, zinc supplementation has been shown to enhance glucose control and insulin sensitivity in patients with diabetes mellitus [29].

In inflammatory conditions, such as inflammatory bowel disease, reduced zinc levels have been found to exacerbate inflammation by leading to the production of inflammatory cytokines and disrupting immune function, thereby worsening disease severity and impairing mucosal healing [30].

In cancer, several studies have discussed the strong correlation between zinc levels and various types of cancers, suggesting that zinc could serve as a potential biomarker for cancer [31,32]. Zinc imbalance can promote tumour development and progression through different mechanisms, including effects on cell proliferation [2], metastasis [33], and apoptosis [34]. The role of zinc in cancer is complex and depends on the cancer type, stage, and the specific molecular mechanisms involved. Serum zinc levels are typically decreased in patients with various types of cancer, including prostate [35], pancreatic [36], lung [37], and breast cancers [38], reflecting increased zinc uptake by tissues for tumour growth. However, zinc levels showed variable patterns in tumour tissues compared to the corresponding tissues. For example, several studies have demonstrated decreased zinc levels in prostate cancer compared with normal tissue [39]. In contrast, elevated zinc levels have been correlated with the progression of some cancer tissues, including breast [40], colorectal [41], and pancreatic cancers [42]. This alteration in zinc levels in cancer is thought to be mediated by the dysregulated expression and function of zinc transporters.

Several studies have shown that the altered expression of zinc transporters varies across different cancer types, with certain transporters being downregulated and others upregulated [42,43]. This alteration in zinc transporter expression could affect the distribution of zinc in cellular compartments, thereby promoting cancer-related cellular processes.

Importantly, the prevalence of chronic diseases in Saudi Arabia, such as diabetes and inflammatory conditions [44] and cancer [45], which are closely linked to zinc dysregulation, is notably high. According to a national systematic review, the incidence of cancer in the Kingdom increased steadily from 2010 to 2019, reflecting a sustained rise in the disease burden [46]. Public health experts have emphasised the urgent need for a comprehensive cancer control strategy in Saudi Arabia, particularly in light of projected increases in cancer-related mortality and healthcare costs [47]. Likewise, the prevalence of diabetes in Saudi Arabia remains among the highest globally, with recent national survey data estimating that over 18% of the adult population is affected, with significant regional variability and high levels of undiagnosed cases [48]. These trends highlight the importance of prioritising local research efforts to explore mechanistic links, such as those involving zinc metabolism and transporter expression, which may help improve disease prevention, early detection, and treatment outcomes tailored to the Saudi population.

Given the established relationships between zinc imbalance, diabetes, inflammation, and cancer, this underscores the urgency of evaluating zinc levels and zinc transporter function, specifically in Saudi patients. Further investigation of zinc dysregulation may reveal novel biomarkers and therapeutic targets relevant to the Saudi healthcare system and public health strategies.

## 4. The Role of LIV-1 Subfamily Zinc Transporters in Cancer Progression

Zinc transporters have been shown to play a vital role in cancer progression by mediating intracellular zinc [22,28,31], highlighting their potential as biomarkers for different cancer types. Multiple studies have demonstrated elevated cellular zinc levels with alterations in zinc transporters across various cancer types, such as pancreatic [42], lung [49], prostate [50], colon [51], and breast cancer [52]. Free cellular zinc in cancer has gained attention because of its correlation with the progression of the disease. In this context, zinc homeostasis is tightly regulated by zinc transporters (ZIP and ZnT family) that control cellular zinc distribution and excretion [53].

**Figure 2:**
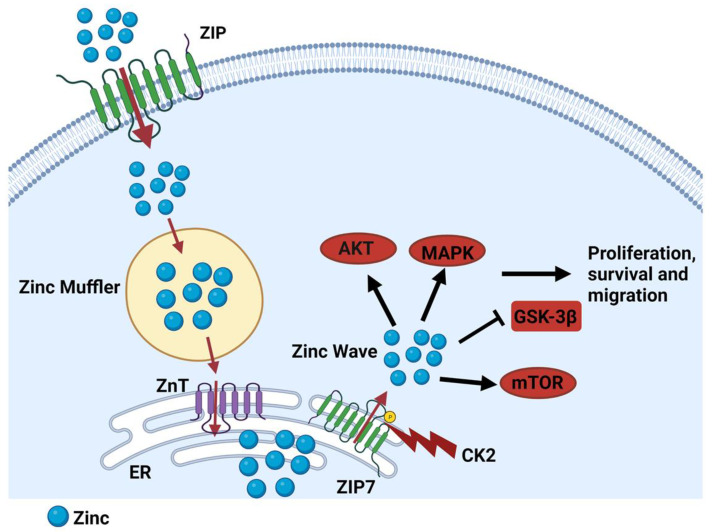
Schematic diagram of ZIP7-mediated zinc release. ZIP transporters import zinc from the extracellular space into the cytoplasm, where zinc is immediately buffered by zinc mufflers, such as MT. Zinc is then stored in the endoplasmic reticulum by ZnTs. After phosphorylation by CK2, ZIP7 releases zinc into the cytoplasm [54]. Subsequently, the released zinc inhibits some tyrosine phosphatases while leading to activate cell proliferation and migration [55].

Imbalances in cellular zinc homeostasis due to alterations in ZIP transporters have been implicated in cancer progression. Among them, ZIP7 (SLC39A7) is located on the endoplasmic reticulum and plays a crucial role in the release of zinc from intracellular compartments [54]. ZIP7-mediated zinc release activates multiple signalling pathways, including MAPK, PI3K, and mTOR, which drive cell proliferation and survival (Figure 2) [55]. ZIP7 has been implicated in various cancers, including breast [52], colorectal [56], and prostate cancer [57]. In breast cancer, ZIP7 is often upregulated, particularly in aggressive subtypes of the disease. ZIP7 overexpression leads to increased intracellular zinc levels, which activate growth factor signalling pathways, promoting cell proliferation and survival. ZIP7 can be activated by phosphorylation by CK2 (casein kinase 2), leading to zinc release from the endoplasmic reticulum and activation of downstream signalling pathways [54,55]. In colorectal cancer, ZIP7 expression is also increased compared to that in normal colon tissue. ZIP7 overexpression is associated with increased cell proliferation, migration, invasion, and resistance to apoptosis [56]. This is supported by the knockdown of ZIP7 in colorectal cancer cells, which diminishes cell proliferation and enhances sensitivity to chemotherapeutic agents [58].

Additionally, ZIP6 (SLC39A6) is located on the plasma membrane and regulated by estrogen; therefore, it is commonly overexpressed in estrogen receptor-positive breast cancer [54]. The upregulation of ZIP6 has been shown to correlate with larger tumour size and lymph node metastasis. ZIP6 facilitates epithelial-to-mesenchymal transition (EMT) through zinc influx and STAT3 activation, thereby promoting cancer cell invasion and metastasis [59]. In colorectal cancer, ZIP6 expression is often upregulated, promoting increased proliferation, migration, and resistance to apoptosis [51]. Similar patterns have been observed in prostate cancer, where ZIP6 is upregulated and linked to aggressive cancer behaviour [60]. Across these cancers, ZIP6 appears to enhance progression by modulating zinc-dependent signalling pathways relevant to cell proliferation.

Similarly, ZIP10, also located on the plasma membrane, is a significant zinc transporter that influences tumour cell behaviour. ZIP10 has been implicated in various cancers, including breast [61], hepatocellular [62], and gastric cancers [63]. In breast cancer, ZIP10 is frequently upregulated, particularly in aggressive subtypes, similar to ZIP6. Consequently, ZIP10 promotes EMT and cancer cell motility through its interaction with ZIP6. ZIP10 and ZIP6 can form a heteromer that plays a key role in EMT and tumour cell migration [61]. In liver cancer, ZIP10 is upregulated and contributes to increased proliferation, migration, invasion, and resistance to apoptosis [62]. While ZIP10’s role in gastric cancer is less defined, a study reported altered expression compared to normal tissue [63]. It may influence gastric cancer progression by regulating intracellular zinc distribution and modulating the related signalling pathways.

Finally, ZIP4 (SLC39A4) has emerged as a critical regulator of zinc homeostasis with significant implications for cancer biology [53]. Originally identified for its role in acrodermatitis enteropathica, a rare genetic disorder characterised by zinc malabsorption, ZIP4 is primarily expressed in the gastrointestinal tract, where it facilitates dietary zinc absorption [64].

Recently, ZIP4 has been shown to be expressed in multiple tumours, particularly pancreatic cancer [65], hepatocellular carcinoma (HCC) [66], and non-small-cell lung cancer (NSCLC) [67]. Its overexpression is associated with enhanced tumour growth, invasion, and resistance to therapy, making it a potential biomarker and therapeutic target. Interestingly, emerging evidence suggests that ZIP4 may also play a role in breast [68] and colorectal cancers [69], although these mechanisms remain less characterised than those of other zinc transporters, such as ZIP6, ZIP7, and ZIP10.

Collectively, the altered expression of zinc transporters in cancer may have important clinical implications for prognosis, as overexpression is often linked to poor outcomes in these cancer types. These transporters also represent potential therapeutic targets, as modulating their activity, such as by inhibiting ZIP7 [70], can reduce cancer cell proliferation and enhance treatment sensitivity.

While the significant role of zinc transporters in cancer progression is globally recognised, their implications in Saudi Arabia have yet to be fully established. Cancer incidence represents a significant health burden in Saudi Arabia [45], and several cancer types have been linked to dysregulated zinc transporters, as previously mentioned. Given the substantial local impact of cancer, research focused on elucidating the expression profiles and clinical significance of these zinc transporters among Saudi cancer patients could uncover population-specific therapeutic targets, potentially improving local cancer management strategies and patient outcomes.

## 5. Cancer Incidence in Saudi Arabia

**Figure 3:**
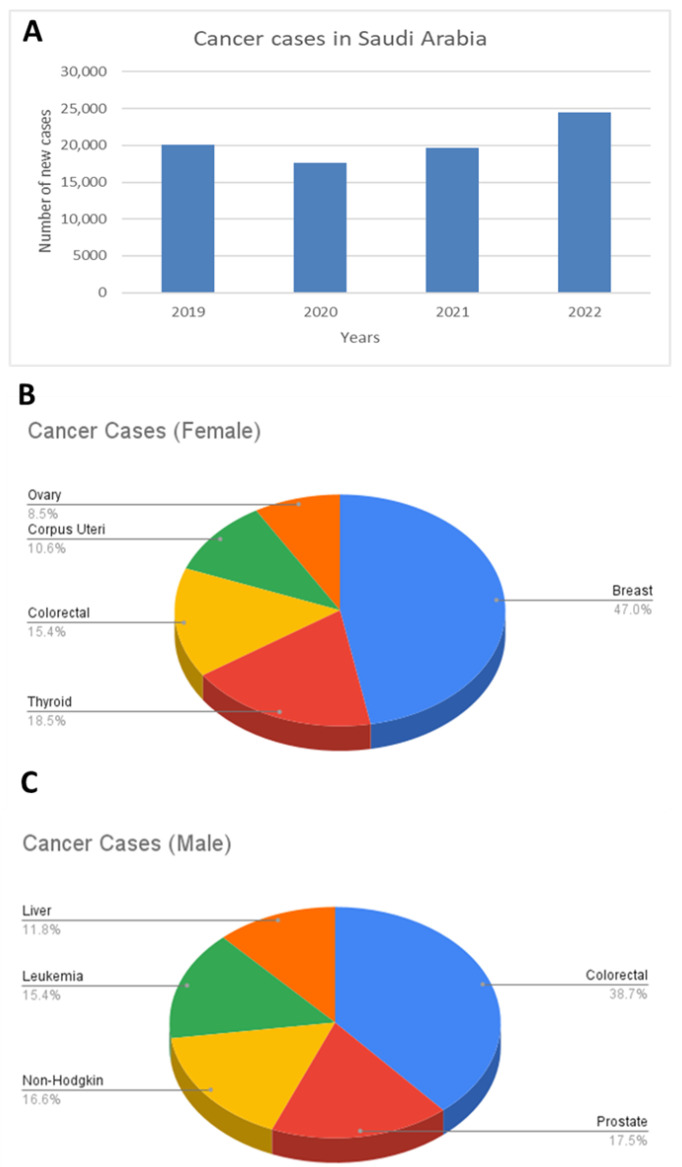
Cancer incidence in Saudi Arabia: trends and gender distribution. (**A**) Annual number of new cancer cases in Saudi Arabia from 2019 to 2022, showing an overall increase over time [71]. (**B**) Distribution of cancer types among females, highlighting breast cancer as the most common, followed by thyroid and colorectal cancers. (**C**) Distribution of cancer types among males, with colorectal cancer being the most prevalent, followed by prostate cancer and non-Hodgkin lymphoma.

To effectively address the role of zinc in cancer within a local context, it is essential to first identify the most prevalent cancers in Saudi Arabia, as this helps guide research priorities and inform public health strategies. Cancer continues to pose a growing challenge nationwide, with the total number of new cases rising to 24,470 in 2022 (Figure 3A). This reflects a sharp 38.8% increase compared to 2020 and a 24.6% increase compared to 2021, reinforcing the post-pandemic rebound in case detection and the growing burden of noncommunicable diseases in the Kingdom. These data were obtained from the Cancer Incidence Report 2022–Saudi Arabia, published by the National Cancer Center, Saudi Health Council, and are available at: https://shc.gov.sa/en/NCC/Activities/Pages/NewAR.aspx (accessed on 9 May 2025) [71].

In 2022, Saudi females accounted for 13,214 cancer cases (54%), whereas Saudi males accounted for 11,256 cases (46%). Among females, breast cancer was the most common, comprising 31.3% of all female cases, followed by thyroid cancer (12.3%) and colorectal cancer (10.2%) (Figure 3B). In males, colorectal cancer was the leading type at 16.8%, followed by prostate cancer (7.6%) and non-Hodgkin lymphoma (7.2%) (Figure 3C). Although the overall age-standardised cancer incidence rates (ASRs) in Saudi Arabia (159.1 per 100,000 in females and 144.9 per 100,000 in males) remain lower than in many European countries such as the United Kingdom (292.5 for females and 327.7 for males) [72], the cancer incidence in Saudi Arabia has shown a consistent upward trend over the past decade. This upward trend reflects how the distribution of cancer in Saudi Arabia is increasingly shaped by modifiable risk factors, including obesity, physical inactivity, and dietary habits [73,74], all of which affect metabolic health and may influence micronutrient status, such as zinc.

Among these, dietary zinc deficiency is an underexplored yet plausible contributor to cancer susceptibility, especially given the essential role of zinc in immune regulation, inflammation control, and cellular signalling. Despite substantial global evidence linking zinc transporter dysregulation to cancer progression, research in this area remains markedly limited in Saudi Arabia. There is a clear gap in understanding how regional genetics, environmental exposures, and culturally influenced dietary patterns, particularly low zinc intake, may contribute to cancer pathogenesis in the local population. Addressing this knowledge gap is critical for developing regionally appropriate prevention strategies and zinc-targeted therapies tailored to Saudi Arabia’s unique demographic and clinical landscape.

## 6. Gene Expression of LIV-1 Subfamily ZIP Transporters in Breast and Colorectal Cancers, the Most Common Forms of Cancer in Saudi Arabia

To investigate the prognostic significance of zinc transporters in cancers that are highly prevalent in Saudi Arabia, the GEPIA 2 server [75] was used to compare the mRNA expression levels of selected ZIP transporters in breast cancer (females) and colon cancer (males), which are the most commonly diagnosed cancers in the Saudi population. These selections reflect both the national cancer burden and the need to align bioinformatic insights with regionally relevant clinical priorities. Tumour versus normal tissue expression was compared for breast cancer and colon adenocarcinoma, focusing on members of the LIV-1 subfamily of ZIP transporters (ZIP4, ZIP6, ZIP7, and ZIP10). All findings were based on gene expression data obtained from The Cancer Genome Atlas (TCGA) and Genotype-Tissue Expression (GTEx) databases. The data were filtered to represent only the differential gene expression between tumours and corresponding normal tissues. A statistical threshold of *p* < 0.01 was applied to identify significantly differentially expressed genes.

**Figure 4:**
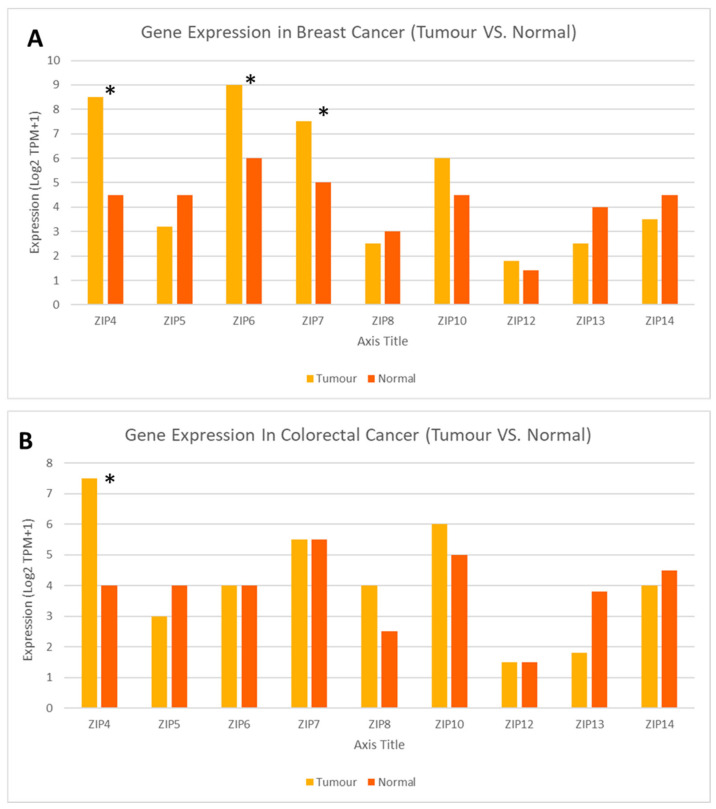
Differential expression profiles of the LIV-1 subfamily of ZIP transporters in tumour vs. normal tissues in breast and colorectal cancer. Expanded analysis of LIV-1 subfamily members of ZIP family transporters in matched normal versus tumour samples from patients with breast (**A**) and colorectal (**B**) cancers. Created using GEPIA2 http://gepia2.cancer-pku.cn/#index (accessed on 9 May 2025) [75]. An asterisk (*) indicates statistical significance at *p* < 0.01.

An expanded gene expression analysis was conducted for all human members of the LIV-1 subfamily of ZIP transporters, given their established involvement in cancer biology [22,25,26] (Figure 4). This approach enabled the identification of key transporters based on differential expression patterns across breast (Figure 4A) and colorectal (Figure 4B) cancers, which represent the most common malignancies in Saudi Arabia. Then, four zinc transporter genes—ZIP4 (SLC39A4), ZIP6 (SLC39A6), ZIP7 (SLC39A7), and ZIP10 (SLC39A10)—were selected based on their documented roles in cancer-related signalling pathways, such as MAPK, PI3K/AKT, and epithelial-to-mesenchymal transition (EMT). In particular, ZIP4 has recently been suggested to play a role in promoting breast and colon cancer tissue proliferation and migration [69]. Similarly, ZIP7 is known to release zinc from intracellular stores and activate proliferative signalling [55], whereas ZIP6 and ZIP10 are associated with cell migration and metastasis, especially in hormone-responsive tumours [59,61].

**Figure 5:**
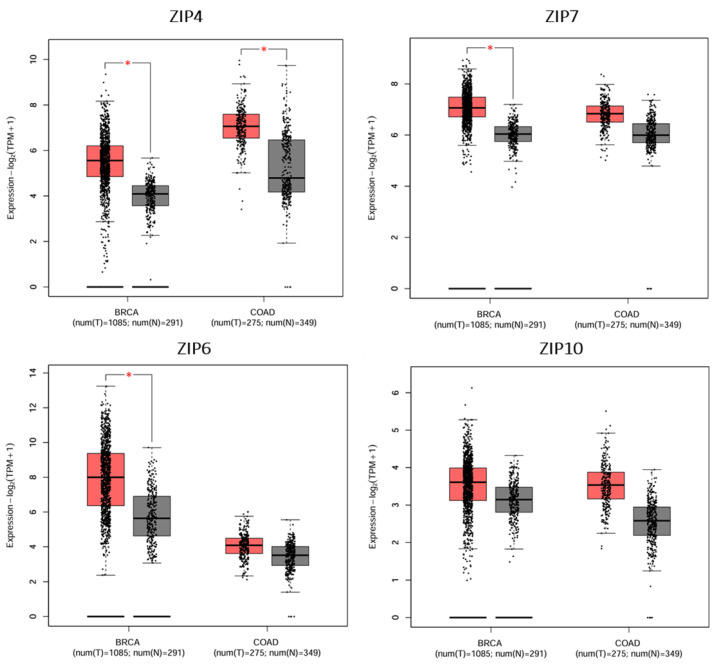
Differential expression profiles of selected ZIP transporters in tumour vs. normal tissues in breast and colorectal cancer. The GEPIA2-generated boxplots of selected ZIP transporters in breast cancer (BRCA) and colorectal cancer (COAD) tumour (T) and normal (N) tissues were based on TCGA RNA-seq data [71]. Grey and red boxes represent normal and cancerous tissues, respectively. All data were based on matched gene expression data obtained from the TGCA and GTEx databases. Significant overexpression of ZIP4, ZIP7, and ZIP6 was observed in breast cancer (BRCA) tumour tissues compared to normal tissues (*p* < 0.05). In colorectal cancer (COAD), only ZIP4 showed significant upregulation in tumour samples. Created using GEPIA2 http://gepia2.cancer-pku.cn/#index (accessed on 9 May 2025) [75]. An asterisk (*) indicates statistical significance at *p* < 0.01.

Expression analysis revealed that ZIP4, ZIP7, and ZIP6 exhibited significant differences between normal and tumour breast tissues, all of which displayed a substantial increase in tumour tissues (*p* < 0.01). In contrast, ZIP10 exhibited a tendency to increase in tumour samples, but the difference was not significant (Figure 5). In particular, ZIP4 was significantly overexpressed in both breast and colon cancers, aligning with emerging evidence of its role in tumour proliferation, invasion, and zinc-mediated oncogenic signalling. Similarly, ZIP7 was significantly upregulated in breast cancer, consistent with its known role in promoting proliferative signalling through zinc-mediated activation; however, no significant difference in ZIP7 expression was observed in colorectal cancer.

Notably, ZIP6 showed marked overexpression in breast cancer and a trend toward increased expression in colon cancer, supporting its involvement in epithelial-to-mesenchymal transition and hormone-responsive tumour progression. Given the fact that ZIP6 and ZIP10 function as a heteromeric complex [61], the observation that ZIP6 was significantly increased in many different tumour tissues, whereas the levels of ZIP10 were only slightly increased, suggests that ZIP6 may be more dominant than ZIP10 in this process. Collectively, these findings highlight the relevance of ZIP transporter dysregulation in the most common cancer types affecting the Saudi population and provide a molecular basis for their potential prognostic and therapeutic values.

## 7. Prognostic Relevance of LIV-1 Subfamily ZIP Transporters in Breast and Colorectal Cancers, the Most Common Forms of Cancer in Saudi Arabia

Having demonstrated the upregulation of ZIP4, ZIP7, and ZIP6 in matched tumour samples, the Kaplan–Meier plotter was used to examine how these expression changes translate to prognosis and disease progression. The Kaplan–Meier plotter is a publicly available online database that evaluates the impact of more than 50,000 genes on the clinical outcome of patients with 21 distinct types of cancer, such as breast and colon cancers [76]. Survival information was obtained from TCGA, Gene Expression Omnibus (GEO), and European Genome-Phenome Atlas (EGA).

Data on ZIP4 (219215_s_at), ZIP7 (202667_s_at), and ZIP6 (202088_at) expression in breast or colon cancer tumour samples were obtained using Affymetrix microarray technology to evaluate messenger RNA (mRNA). JetSet-optimised probes were used for ZIP4, ZIP7, and ZIP6.

The cut-off value of gene expression was determined using the auto-select best cut-off method, which divided the patient samples into two groups to generate the corresponding plots. Survival analyses were conducted to evaluate the impact of ZIP4, ZIP7, and ZIP6 expression on both overall survival (OS) and relapse-free survival (RFS) in breast and colon cancers. This dual analysis provided insights into long-term survival outcomes and the likelihood of disease recurrence. Log-rank *p*-values were calculated using the Kaplan–Meier plotter interface, with *p* < 0.05 considered statistically significant. Survival analyses were performed only for ZIP transporters that demonstrated significant overexpression in specific cancers based on expression profiling. Specifically, ZIP4 was analysed in both breast and colon cancers, whereas ZIP7 and ZIP6 were analysed exclusively in breast cancer. ZIP10 was excluded from this section due to its expression pattern in tumour tissues.

**Figure 6:**
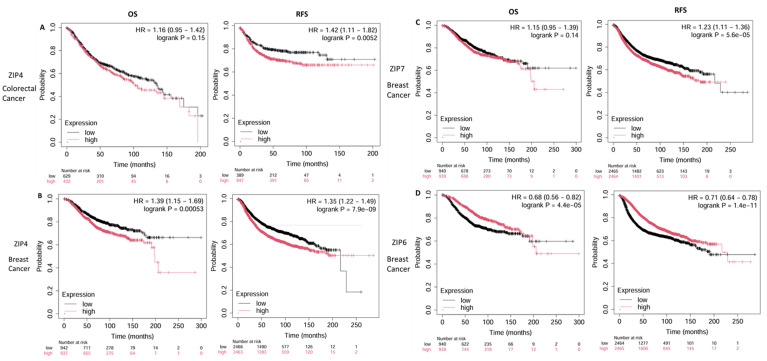
Kaplan–Meier survival analyses of ZIP transporter expression in breast and colorectal cancer. Kaplan–Meier survival plots illustrating the impact of high (red) versus low (black) ZIP transporter expression on overall survival (OS) and relapse-free survival (RFS) in patients with breast cancer (BRCA) and colorectal cancer (COAD). Survival data were obtained from the Kaplan–Meier plotter tool using TCGA and other publicly available datasets. High ZIP4 expression was associated with significantly poorer RFS in both colorectal (**A**) and breast cancers (**B**) (HR and *p*-values are shown in each plot). ZIP7 (**C**) and ZIP6 (**D**) were analysed only in breast cancer because of their significant overexpression in this cancer type, where ZIP7 showed a negative impact on OS and RFS, whereas ZIP6 was linked to improved outcomes. Created using Kaplan–Meier plotter: https://kmplot.com/analysis/ (accessed on 9 May 2025) [76].

In colon cancer, elevated ZIP4 expression was significantly associated with poorer RFS, whereas the trend toward worse OS did not reach statistical significance, possibly due to sample size or other confounders (Figure 6A). Patients with high ZIP4 expression had a median RFS of approximately 32.6 months, compared with 116 months in those with low ZIP4 expression levels.

However, the difference in OS between the high- and low-expression groups was not statistically significant, with median OS values of 102 and 135 months, respectively. These results suggest that ZIP4 may be more strongly associated with disease recurrence rather than long-term survival, pointing toward a possible role in therapy resistance or early progression.

In breast cancer, high ZIP4 expression was strongly associated with poorer OS and RFS (Figure 6B). Patients with elevated ZIP4 expression had an observed reduced OS compared to those with low expression. Similarly, ZIP4 overexpression was associated with a significantly lower RFS. These findings indicate that ZIP4 may serve as a negative prognostic marker in breast cancer, potentially contributing to disease progression and early recurrence.

Similarly, elevated ZIP7 levels in patients were strongly associated with poor prognosis (Figure 6C). Patients with high levels of ZIP7 tend to have lower rates of OS (82 months) and RFS (185 months), compared to 106.8 and 216.66 months, respectively. These findings suggest that high ZIP7 expression is associated with a poorer prognosis and could potentially be a mechanism for the development of resistance.

In contrast, ZIP6 expression was significantly positively associated with prognosis, with patients with high ZIP6 levels showing increased OS (125.92 months) and RFS (65 months), compared to 63.52 and 28.96 months, respectively (Figure 6D). These findings indicate that ZIP6 is associated with a better prognosis in patients with breast cancer. These improved outcomes may reflect the potential role of ZIP6 in maintaining cellular differentiation, moderating oncogenic signalling, and enhancing sensitivity to chemotherapy. Therefore, it could potentially be a biomarker for good prognosis in breast cancer.

## 8. Discussion

This review examined the complex relationship between zinc transporters and cancer progression, with a particular focus on their expression patterns and prognostic significance in common cancers prevalent in Saudi Arabia. Bioinformatic analysis revealed an upregulation of ZIP4, ZIP7, and ZIP6 gene expression in tumour samples compared to the corresponding normal tissues, while ZIP10 showed a non-significant increasing trend. Notably, elevated tumour expression of these transporters was associated with poorer survival outcomes. These findings provide a clear molecular basis for the dysregulation of zinc homeostasis in cancer and highlight the potential role of these transporters as prognostic biomarkers and therapeutic targets in cancer.

The varied expression patterns of these zinc transporters reflect their unique contributions to cancer biology. For example, ZIP4 is significantly overexpressed in both breast and colon cancers, showing a distinct role in cancer progression beyond what was previously recognised. Similarly, ZIP7 was consistently upregulated in breast cancer, highlighting its potential role as a mediator of zinc-dependent oncogenic signalling in this cancer type. This supports its established role in the release of zinc from intracellular stores to activate the growth factor pathways [54,55]. However, ZIP7 expression was not significantly elevated in colorectal cancer using TCGA/GTEx RNA-seq data via GEPIA2. This finding contrasts with Luo et al. [56], who reported elevated ZIP7 expression in colorectal cancer using immunohistochemistry in a single-centre cohort with long-term clinical follow-up. Differences in assay type, whether based on protein measurement (immunohistochemistry) or RNA sequencing, along with tumour stage or patient population, may explain this discrepancy. ZIP6 showed marked upregulation, particularly in breast cancer, consistent with its known involvement in epithelial-to-mesenchymal transition and metastasis [59]. The prognostic analysis further revealed that these expression patterns translated to clinical outcomes, with high ZIP4 and ZIP7 expression generally associated with poorer outcomes, whereas ZIP6 showed a positive association with prognosis in breast cancer.

This review contributes to the existing knowledge of ZIP transporters in cancer, particularly ZIP4, which has limited studies in breast and colorectal cancer compared to pancreatic and hepatocellular carcinomas [65,66]. These results are consistent with existing evidence suggesting ZIP4’s role extends beyond its well-recognised functions in zinc absorption in the gastrointestinal system. ZIP4 is primarily involved in zinc uptake from the gastrointestinal tract [64], helping to regulate overall zinc homeostasis by transporting zinc into the cell, where it is stored in intracellular compartments and later mobilised by ZIP transporters. In cancer, the increased use of zinc stores results in zinc deficiency within tumour cells, leading to the upregulation of ZIP4 to restore intracellular zinc levels. This compensatory upregulation of ZIP4 in tumour cells, driven by increased intracellular zinc demand and depletion of zinc stores, may also help explain the observed serum zinc deficiency in patients with cancer [35,37,77].

Recent studies have shown that ZIP4 upregulation in pancreatic cancer promotes the IL-6/STAT3 signalling pathway and activates the production of neuropilin-1 and vascular endothelial growth factor (VEGF), thereby enhancing tumour growth and angiogenesis [78]. In a similar manner, overexpressed ZIP4 levels in hepatocellular carcinoma have been associated with elevated expression of matrix metalloproteinases, facilitating extracellular matrix breakdown and invasion [33]. In this review, the findings on breast and colorectal cancers suggest that comparable oncogenic mechanisms may be involved, although the specific pathways require further investigation.

The noticed association between ZIP4 overexpression and poorer relapse-free survival in both breast and colorectal cancers, along with decreased overall survival in breast cancer, indicates its potential as a prognostic marker. This finding aligns with studies on pancreatic cancer, where elevated ZIP4 levels are linked to advanced disease stages and poor prognosis [42,79].

Similarly, in colorectal cancer, the observed correlation with relapse-free survival compared to overall survival indicates that ZIP4 may play a specific role in disease recurrence [80], possibly through mechanisms related to therapeutic resistance or early progression.

Taken together, these findings strongly support the emerging view that zinc transporters are not only involved in metal ion mobilisation but also act as key regulators of signalling pathways critical to cancer progression.

The contrasting prognostic implications of ZIP6 and ZIP7 indicate the context-dependent role of zinc transporters in cancer. While upregulated ZIP7 is correlated with poorer patient outcomes in breast cancer, aligning with its function in promoting oncogenic pathways [55], ZIP6 exhibited a positive correlation with survival in breast cancer.

Notably, this association was observed in a cohort of patients treated with chemotherapy, suggesting that higher ZIP6 expression may be linked to less aggressive tumour behaviour. This may reflect the fact that ZIP6 is regulated by oestrogen and is frequently overexpressed in oestrogen receptor-positive breast cancers, which are typically associated with a better prognosis [59]. However, ZIP6 has been shown to mediate zinc influx, driving cell division [81]. Thus, the observed improvement could be attributed to an increased sensitivity to chemotherapy, which might be due to the accelerated growth rates of tumours exhibiting high ZIP6 levels.

The dysregulation of zinc transporters in cancer has significant diagnostic implications for cancer treatment. Zinc transporters, such as ZIP4 and ZIP7, have emerged as potential prognostic biomarkers, with elevated expression levels associated with more aggressive diseases and poorer clinical outcomes. Measuring their expression in patients with cancer could help develop appropriate personalised treatment strategies. For example, high levels of ZIP4 or ZIP7 in breast cancer tissues may indicate a greater risk of recurrence or metastasis, whereas overexpression of ZIP4 in colorectal cancer has been linked to reduced survival, as demonstrated by both this bioinformatic analysis and external clinical studies [80].

This finding has fundamental implications for Saudi Arabia’s healthcare system. With breast cancer representing 31.3% of female cancer cases and colorectal cancer comprising 16.8% of male cases in the Kingdom, and their incidence having risen dramatically (by approximately 10-fold between 1990 and 2016) [45], analysing zinc transporter expression could play a crucial role in improving risk assessment and treatment planning for a significant proportion of the cancer burden.

Incorporating zinc transporter profiling into diagnostic workflows could provide valuable prognostic information, particularly for ZIP4 and ZIP7, which have shown consistent associations with poorer outcomes. This strategy aligns with the Saudi Vision 2030 healthcare transformation plan, which prioritises precision medicine and innovative diagnostic methods. This is particularly important in light of the widespread micronutrient deficiencies reported among Saudi adults, the predominance of phytate-rich dietary patterns that may impair zinc absorption, and the notable lack of local research on zinc transporter expression in cancer.

The altered expression of zinc transporters in tumours significantly impacts zinc homeostasis in the body. Zinc is an essential trace element for cellular growth and development, and its cellular levels fluctuate in response to cancer. Herein, the findings suggest that the elevated expression of zinc transporters in tumours promotes zinc uptake by cancer cells, consequently depleting zinc levels in the bloodstream. This observation is consistent with previous clinical studies that reported lower serum zinc levels in patients with cancer than in healthy individuals. For example, a large meta-analysis found that patients with breast cancer exhibit significantly lower serum zinc levels than healthy controls [38]. A similar pattern of reduced circulating zinc levels has been observed in patients with colorectal cancer [77]. Mechanistically, cancer cells may uptake zinc to support DNA synthesis, anti-apoptotic proteins, and rapid growth, thereby altering zinc redistribution, leading to increased zinc content in tumour tissue and reduced availability in the bloodstream. This inverse correlation significantly highlights the role of malignant tissue in sequestering zinc from circulation.

The frequently observed reduction in serum zinc levels among cancer patients may be attributed to the altered expression of zinc transporters, particularly ZIP4 and ZIP7, which facilitate increased zinc uptake and release within cancer cells, respectively. A striking example has been observed in colorectal cancer, where ZIP4 expression is positively correlated with cancer stage and a concurrent decline in serum zinc levels [76,80]. Similarly, ZIP7 is significantly upregulated in aggressive breast cancer subtypes [52], suggesting its role in disease progression and potential resistance to therapy. Locally, a study conducted on patients with prostate cancer in Saudi Arabia found decreased levels of trace elements, including zinc, compared with healthy controls [35]. Although prostate cancer differs from colorectal and breast cancers in terms of tissue origin, the findings suggest a shared pathophysiological mechanism involving disrupted zinc homeostasis. This mechanistic understanding enhances the interpretative value of serum zinc levels and suggests that the combined assessment of serum zinc and transporter expression profiles could provide more comprehensive diagnostic information for oncology and patient care.

The relevance of these findings for Saudi Arabia extends beyond diagnostic implications to include therapeutic interventions. The significant relationship between the upregulation of ZIP4 and ZIP7 and poor clinical outcomes indicates that these transporters are promising therapeutic targets. Potential strategies include the development of antibodies to block their activity and modulate their function, RNA interference techniques to suppress their expression, or small-molecule inhibitors to prevent zinc uptake. Notably, preclinical research has shown that silencing ZIP7 or using an emerging ZIP7 inhibitor can inhibit tumour growth and metastasis in cancer models [34,82]. Given the similar oncogenic roles of ZIP4, these strategies could also be applicable to ZIP4, providing a potential strategy for targeted therapies in cancers such as breast and colorectal cancer, which are highly prevalent in the Saudi population.

## 9. Limitations and Future Directions

Despite these promising findings, several limitations must be acknowledged. First, although the analysis revealed a relationship between zinc transporter expression and cancer outcomes, the underlying mechanisms, particularly those involving ZIP4 in breast and colorectal cancer, remain unclear. Second, clinical studies using patient-derived samples from the Saudi population are needed to elucidate these mechanisms, as environmental factors and local genetic backgrounds may affect zinc transporter activity compared to other populations. Third, the bioinformatic analysis obtained in this review was based on publicly available datasets, which may not effectively reflect the environmental and genetic diversity that is unique to Saudi Arabia. Therefore, population-specific research is required to validate these findings in the Saudi context.

While this review uses global datasets, future research should include a comprehensive profiling of zinc transporters in Saudi cancer patients, incorporating genomic and proteomic approaches to fully investigate the disruption of zinc homeostasis. Furthermore, functional studies focusing on elucidating the mechanisms through which ZIP4 and ZIP7 contribute to the progression of breast and colorectal cancers would provide critical insights into the development of targeted therapeutic strategies.

The relationship between dietary zinc intake and zinc transporter expression in the Saudi population requires further investigation. Cultural dietary patterns may influence zinc status and transporter function, potentially contributing to regional variations in cancer incidence and outcomes. Given the fact that zinc plays a critical role in supporting immune function, inadequate dietary zinc intake may impair the immune response, potentially worsening cancer prognosis. Understanding these relationships can inform nutritional guidelines and public health interventions tailored to the Saudi population.

## 10. Conclusions

In summary, this review highlights the differential expression and clinical relevance of LIV-1 subfamily zinc transporters in breast and colorectal cancer, which represents a major portion of the cancer burden in Saudi Arabia and globally. ZIP4 was significantly upregulated in both cancer types and associated with worse relapse-free survival. In contrast, ZIP7 showed consistent overexpression only in breast cancer and was associated with poorer patient outcomes, reinforcing its role as a mediator of zinc-dependent oncogenic signalling in this context. The distinct expression patterns correlated with clinical outcomes highlight their potential as both diagnostic biomarkers and therapeutic targets. Analysing zinc transporter expression could enhance risk assessment and guide treatment planning broadly, while also informing research strategies relevant to the Saudi population. Moreover, therapeutic interventions targeting these transporters provide a promising direction for cancer therapy, which aligns with the Saudi Vision 2030. However, further research on zinc homeostasis in cancer is essential for understanding new strategies to improve patient care and outcomes, both nationally and globally.

## Data Availability

The data will be made available upon request. The data supporting the findings of this study are available within the paper. Should any raw data files be needed, they are available from the corresponding author upon reasonable request.
